# The vicious interplay between disrupted sleep and malignant brain tumors: a narrative review

**DOI:** 10.3325/cmj.2021.62.376

**Published:** 2021-08

**Authors:** Darko Orešković, Anđelo Kaštelančić, Marina Raguž, Domagoj Dlaka, Nina Predrijevac, Dinko Matec, Martina Matec, Damir Tomac, Vjekoslav Jeleč, Tonko Marinović, Darko Chudy

**Affiliations:** 1 Department of Neurosurgery, Dubrava University Hospital, Zagreb, Croatia; 2 Croatian Institute for Brain Research, Zagreb University School of Medicine, Zagreb, Croatia; 3 Department of Traumatology and Orthopedics, Dubrava University Hospital, Zagreb, Croatia; 4 Department of Oncology and Radiotherapy, University Hospital Center, Zagreb, Croatia; 5 Department of Neurology and Neurosurgery, Faculty of Dental Medicine and Health, Josip Juraj Strossmayer University of Osijek, Osijek, Croatia; 6 Department of Surgery, Zagreb University School of Medicine, Zagreb, Croatia

## Abstract

Malignant brain tumors are among the most aggressive human neoplasms. One of the most common and severe symptoms that patients with these malignancies experience is sleep disruption. Disrupted sleep is known to have significant systemic pro-tumor effects, both in patients with other types of cancer and those with malignant brain lesions. We therefore provide a review of the current knowledge on disrupted sleep in malignant diseases, with an emphasis on malignant brain tumors. More specifically, we review the known ways in which disrupted sleep enables further malignant progression. In the second part of the article, we also provide a theoretical framework of the reverse process. Namely, we argue that due to the several possible pathophysiological mechanisms, patients with malignant brain tumors are especially susceptible to their sleep being disrupted and compromised. Thus, we further argue that addressing the issue of disrupted sleep in patients with malignant brain tumors can, not just improve their quality of life, but also have at least some potential of actively suppressing the devastating disease, especially when other treatment modalities have been exhausted. Future research is therefore desperately needed.

The annual incidence of tumors of the central nervous system (CNS) is little over 22 per 100 000 in the general population (1). Around a third of these lesions are malignant. Among the malignant tumors, gliomas are by far the most common type, constituting over 80% of the number. Among gliomas, the most aggressive type (glioblastoma) is the most common one, making up over a half of all newly diagnosed gliomas (2,3). The five-year survival of patients with malignant CNS tumors is around 30%, with patients being diagnosed a glioblastoma having a five-year survival rate of less than 5%. All this goes to show how malignant CNS tumors are some of the most aggressive human malignancies today. It also shows how the vast accumulated knowledge on the disease origin and progression still has not translated into significant improvement of the overall survival of these patients. New treatment modalities are therefore desperately needed.

Besides the devastating diagnosis of a malignant brain tumor, these patients often experience a wide variety of severe symptoms, which significantly diminish their quality of life (4). There has been an increasing awareness of the importance of supportive and palliative care in patients suffering from malignant brain tumors, especially those in whom other treatment modalities have been exhausted (5-7). One of the most commonly reported symptoms is sleep disturbance (4,8-12).

Sleep is a recurrent, physiological phenomenon, which consists of many measurable factors (12) and is ubiquitous throughout the natural world (13-16). It is a highly active, easily reversible process, which is crucial not only for the physical and mental well-being of all living organisms, but also for the very concepts we as humans have of ourselves and the world around us (17). There are many theories regarding the possible function of sleep, ranging from the physiological explanations such as rest of individual cells (18) to behavioral explanations of why a biological system needs periodic inactivity (19). There is a growing understanding of how the modern lifestyle disrupts the natural circadian rhythm in humans, consequences of which are still not sufficiently explored (20).

Sleep disruption has a well known detrimental role for an organism. Indeed, patients with disrupted sleep have been found to have a higher prevalence of several diseases, such as cardiovascular disorders (21), cognitive impairment (22), various metabolic disorders and obesity (23,24), and systemic and local inflammation (25,26). Furthermore, sleep can be impaired in many ways. The current classification of sleep disorders consists of several clinical entities such as insomnia, parasomnia, hyper-somnolence, sleep-related movement disorders, etc (27). However, this article refers to all of this broad pathology as “sleep disturbance,” primarily for clarity and simplicity sake. In addition, research on disrupted sleeping patterns in patients with malignant lesions usually also encompasses all of these entities into this broader term (28,29).

## Disrupted sleep in patients with a malignant disease

An emerging field of interest in sleep disruption in patients suffering from malignant diseases has recently gained much attention (11,29). Indeed, it has been shown that disrupted sleep is one of the most common complaints in patients undergoing oncological treatment, with patients suffering from malignant brain tumors being especially susceptible (4,10). Furthermore, the risk of developing several different neoplasms can be directly correlated with various sleep disturbances (30-33). On the other hand, patients suffering from various types of neurological disorders very often also suffer from some sort of disrupted sleeping pattern (34,35), indicating that patients with CNS pathology are very susceptible to sleep disruption.

Research into the complex relationship which malignant brain tumors could have with disrupted sleep is quite scarce. It usually addresses the severity and frequency with which disrupted sleep occurs as a symptom in these patients (8,9,36) as well as the effects of the oncological treatment on sleeping patterns (8), but usually does not explore in detail the possible pathophysiological mechanisms by which disrupted sleep could actually detrimentally affect patients‘ ability to fight off the disease, or the ways in which malignant brain lesions themselves could actually impair sleep.

The aim of this article was therefore to review the currently available research concerning sleep in patients with malignant brain tumors. More specifically, we provide a review of the known consequences of disrupted sleep on the malignant progression and connect it to the currently available research regarding malignant brain tumor pathology. The secondary aim, which is explored in the second part of the article, was to provide theoretical framework for the possible pathophysiological effects that a malignant brain lesion itself could have on the brain’s ability to sleep.

## Systemic effects of disrupted sleep

Alongside other detrimental effects stated above, there have been several proposed mechanisms of the possible pro-tumor effects of disrupted sleep (2,30). As mentioned earlier, however, these reports focus on many other types of malignant diseases, but usually leave out patients with brain tumors, a fact already noted elsewhere (8,28). We will therefore explore several possible mechanisms of the pro-tumor effects of disrupted sleep that have been linked to other malignancies and determine the possible correlation it could have to malignant brain tumors. These mechanisms include phase shifts, reduced anti-oxidant levels, immunosuppression, metabolic changes, melatonin depletion, cognitive impairment, and epigenetic changes. All of these systemic changes have in turn been linked to a worse prognosis in patients with malignant brain tumors, indicating disrupted sleep as a possible exacerbating factor of tumor progression. This connection has been differently proven for various factors and we tried to note them here in descending order, starting from the ones with the most clinical proof.

### Disrupted sleep and phase shift

Disrupted sleeping patterns have been found to significantly influence the transcription of the so-called clock genes (37). These genes, alongside the circadian “master clock” in the suprachiasmatic nuclei of the brain, govern the rhythmic circadian synchronization of almost all of the physiological processes within the body (2).

The physiological circadian clock functions as a tumor suppressor at the systemic, molecular, and cellular levels. Indeed, these circadian rhythms have been found to be so important that their various disruptions lead to the so-called phase shifts, which have been linked to both tumorigenesis and tumor progression (2,38). In fact, there is a growing awareness of how chronotherapy could improve the efficacy of cancer treatment and the quality of patients’ lives (38,39).

Malignant brain tumors have been found to rely heavily on the expression of clock genes, namely in their growth (40), cellular proliferation (41), and migration (42). It is therefore very likely that the disruption of various circadian sleep-wake cycles further impairs the physiological circadian rhythms, thus having crippling pro-tumor effects.

### Disrupted sleep and reduced antioxidant levels

Excess production of free radicals (or oxidative stress) plays an important role in the metabolism of all living aerobic organisms, including humans. These free radicals, also called reactive oxygen species, induce oxidative damage to certain cellular macromolecules, and this damage has been linked to many common human diseases including cancer (43). Several protective cellular mechanisms have evolved to counter this damage, namely in the form of various antioxidant molecules and antioxidant enzymes (44). Of these, glutathione has been found to be the most important mammalian cellular antioxidant molecule, with a crucial role in cell protection against oxidative stress (44).

Glutathione is a well-known antioxidant molecule with a significant protective role against oxidative free-radicals and carcinogens (45). However, glutathione levels are not constant. They actually strongly depend on the circadian rhythm. More specifically, they are significantly elevated during sleep (46). Thus, due to the disruption of sleep cycles, physiologically elevated levels of glutathione are diminished, making cells more susceptible to oxidative damage.

The therapeutic potential of glutathione is highly complex and controversial. Indeed, beside its protective role, glutathione also significantly influences the response to therapy of the tumor cells themselves, namely it allows these cells to suffer less damage from oncological therapy (47,48). Bansal and Simon (45) offer a more in-depth analysis of the immensely complex dual role which glutathione plays in cancer patients. The knowledge of these complex systems and mechanisms is still insufficient, and further research is desperately needed.

Beside glutathione reduction due to the lack of sleep, there is also another mechanism that diminishes glutathione levels and that occurs in patients with malignant brain tumors. It involves glutamate metabolism and is explored in greater detail in the section regarding chemical sleep disruption.

### Disrupted sleep and immunosuppression

It is well-known that patients with malignant brain tumors experience a significant local and systemic immunosuppression (49,50). This is an area of intense recent interest with regard to potential therapeutic options (51). While the exact mechanisms of this suppression are unclear, one possible explanation could be that the tumor alters sleeping patterns, which in turn influences immunosuppression. 

It has been shown that disrupted sleep can impair the systemic immune response both in animals (52) and in humans without (53,54) and with (55) malignant disease. In patients with malignancies, disrupted sleep seems also to negatively affect the immune system, primarily by disrupting the functioning of natural killer cells and cytokine production (55). 

It therefore seems possible that future treatments targeting the immune response of patients will have to account for disrupted sleep as well.

### Disrupted sleep and metabolic changes

Even short-term sleep loss has been shown not only to disrupt the physiological functioning of various metabolic processes such as glucose regulation or cortisol and insulin secretion, but also to lead to an increased appetite and caloric intake (20). Chronic sleep disruption has also been linked to severe complications such as cortisol and insulin dysregulation, obesity, and diabetes mellitus (20,56).

These alterations to various aspects of metabolic syndrome have on the other hand been consistently connected to a worse prognosis in patients with cancer (57), as well as with malignant brain tumors (58,59). Our own preliminary research on patients with meningiomas (60) and patients with glioblastomas (61) showed that at least some patients with primary intracranial malignancies could actually have significantly disrupted chronic blood glucose levels. Even on the cellular level, it has been shown that genes coding for various glucose transporters (GLUT1 for example) are differently expressed in the sleeping brain than in the awake one (46).

It seems therefore likely that the metabolic changes linked with disrupted sleep further impair the metabolic status of patients with brain neoplasms, leading to a worse prognosis.

### Disrupted sleep and melatonin depletion

Melatonin is a pineal hormone that is involved in the circadian regulation and facilitation of sleep (62). Besides in the pineal gland, melatonin is also synthesized in various other organs, tissues, and cells, also in a circadian fashion, with a high rhythm amplitude and a prominent nocturnal maximum. In the extrapineal sites, secretion oscillations have considerably lower amplitudes. Some of the extrapineal sources are, according to current knowledge, of particular importance, either in quantitative terms, such as the gastrointestinal tract, which contains several hundred times more melatonin than the pineal gland or, with regard to functional aspects (specific areas of the central nervous system, white blood cell count, etc).

Melatonin is also often called a hormone of darkness since all of the body's melatonin is secreted at night-time (63). Melatonin has been shown to have various anti-tumor effects, both in malignant brain tumors (64,65) and in other cancers (63,66). Besides this protective role in malignant brain tumor patients, melatonin also has many other possible anti-tumor mechanisms, which link it to points mentioned earlier. These include such diverse effects as the ones on the systemic immune response, its antioxidant role, its effect on glucose regulation, etc (67,68).

The modern, industrialized lifestyle with its dependency on light disrupts significantly the synthesis and the secretion of melatonin (63,69). Even different diets have been found to affect the melatonin levels in the organism (70). It has also been found that disrupted sleeping schedules significantly further diminish melatonin secretion (63,71).

Melatonin levels are therefore likely altered in patients with malignant brain tumors due to their impaired sleeping schedules, thus diminishing the many possible anti-tumor effects of the hormone. And while melatonin is currently successfully administered in the treatment of restoring the diurnal rhythm (72), its complex metabolism has repeatedly been suggested as a possible therapeutic target in oncological treatment (73,74). This type of research is still unfortunately in its infancy and further investigation is needed.

### Disrupted sleep and cognitive impairment

Sleep disruption has been shown to severely diminish the quality of life in patients and their families through various mood disorders, memory impairment, increased dementia risk, etc (20). Cognitive impairment is also known to be connected to a worse survival in patients with malignant brain tumors (75). Therefore, it is possible that disrupted sleep at least partially exacerbates the cognitive impairment of these patients, also leading to a worse prognosis.

### Disrupted sleep and epigenetic changes

There is a growing awareness of the significant effects of sleep disruption on the epigenome (76). Epigenetic changes have on the other hand been found to have a crucial role in the modern understanding of brain malignancies and their classification (77). It is therefore possible that disrupted sleep alters in some way various epigenetic environments in malignant brain tumor patients, a currently underexplored field with significant potential.

## Tumor effects on sleep

Having discussed the possible ways in which disrupted sleep as a common symptom can influence the tumor progression, we will now focus on the effects of the tumor itself on the brain’s ability to sleep.

### Physical effects

#### Direct physical effects

Sleep disturbances are a very common symptom of patients with brain tumors (4,10). The exact causes of these disturbances are still largely unknown, with several articles mentioning various possible explanations (8). The authors here include the patients’ comorbid conditions, concurrent symptoms, environmental stressors, prescribed medication, as well as neuropsychiatric effects. The authors also mention the so-called direct tumor effects, namely pituitary/hypothalamic involvement and anhedonia. We however feel that additional possible mechanisms could be put into the latter category that the authors do not explore. Indeed, we feel that these direct effects of the tumor make patients even more susceptible to subsequent environmental mechanisms mentioned earlier. Although the research into this area has unfortunately been limited, sporadic reports show that sleep could actually be impaired even before chemo- and radiotherapy or other external factors (78). This is also true in our preliminary research (data currently unpublished).

#### Direct disruption in the sleep-wake circuitry

This mechanism is straightforward and has been mentioned in previous research. It seems fairly obvious that a malignant lesion that destroys the neural projections or the structures involved in the sleep-wake circuitry (the hypothalamus for example) would disrupt the sleeping patterns. It has indeed been reported that patients with a malignant tumor in these regions could have severely impaired sleeping cycles (79).

#### Lesions in parts of the brain not directly involved in sleep-wake regulation

The brain function inevitably relies on its structure. And, as mentioned earlier, one of the most important brain functions is sleep. Therefore, it is likely that the complex changes occurring during sleep are not limited to certain cerebral areas, but are instead function of the entire brain (80). It would follow then that any disruption in the cerebral structure can impair sleep to a greater or lesser degree. More specifically, even malignant lesions in the brain regions that are not usually considered to be crucial for sleep could mechanically disrupt the complex cerebral structure and its function. Indeed, sleeping is disrupted in many other neurologic pathological conditions that compromise the general brain structure (35,81). Also of note is that the growing intracranial mass can cause many other non-specific symptoms (such as headaches) during sleep and thus further impairing the sleeping schedule of a patient.

#### Indirect physical effects

A growing intracranial mass can cause a variety of non-specific symptoms. This is especially true for highly proliferative malignant tumors, where the compensatory mechanisms of an organism are rapidly rendered insufficient due to the fast and infiltrative tumor growth. The most commonly reported indirect physical effect of a malignant lesion on brain function is disruption in a patient’s breathing patterns.

Breathing is a highly complex physiological phenomenon. It is tightly controlled in several distinct control points, namely the central control at the level of the brainstem, effector control (respiratory muscles for example), and sensory control. The central breathing control is primarily performed by three large neuron groups in the pons and medulla (82,83). Disruption or damage in these neurons and neuron groups can lead to severe breathing disorders and even death (Ondine’s curse). Beside malignant lesions, such damage can occur in other types of neuropathology as well, such as in multiple sclerosis (84). Breathing is also controlled at a higher level, namely through corticobulbar and corticospinal pathways (82). This supra-pontine control allows for voluntary respiration modification. Destruction of these pathways can also lead to severe breathing disorders (85). Another possible mechanism by which malignant brain tumors can compromise breathing is through elevated intracranial pressure, which arises due to the growing lesion. This in turn can cause an indirect compression of the breathing centers, similarly to what occurs in patients with Chiari malformation (86).

All of the mechanisms described above can compromise breathing and breathing patterns of a patient, causing various types of dyspneas and apneas, especially during sleep. Indeed, it has already been recognized that sleep-disordered breathing has a much higher prevalence in patients with neurologic disorders such as stroke and epilepsy (87). While these sleeping disorders have been labeled highly prevalent and grossly under-recognized (88), they have also not been sufficiently investigated in patients with malignant brain tumors. This seems remarkable since it has already been shown that apneas and insomnias lead to a functional reorganization of the brain (89,90). Moreover, this type of sleep disruption has been connected to a worse outcome and prognosis in several diseases, including malignant ones (91). Furthermore, when different effects of acute, chronic, and cyclic hypoxia were investigated with regards to tumor aggressiveness, it has been shown that cyclic hypoxia (such as the one occurring in patients with sleep apneas) actually significantly enhances tumor cell aggressiveness by altering various cancer hallmarks, such as angiogenesis, metastasis, cell proliferation, and/or inflammation (92). Although these findings have not yet been tested on brain tumor cells, it seems possible that similar effects could take place in them as well.

Knowing how common and often undetected various breathing disorders are in the general population (87,93), and taking into account the pathophysiological mechanisms described above, it is likely that patients with brain tumors are also susceptible to this type of pathology. Unfortunately, research is still fairly limited and warrants further effort into deciphering the complex relationship which brain tumors, sleeping, and breathing have with each other.

### Chemical sleep disruption

Alongside all of the aforementioned mechanisms by which malignant brain tumors can disrupt sleeping patters of a patient through their physical interaction with normal brain tissue, there is also another important way in which this disruption can occur. The disruption in question is a chemical one, through glutamate.

Glutamate is not only the predominant excitatory neurotransmitter in the central nervous system, but it also has a crucial role in regulating sleep-wake cycles (94). More specifically, it has a significant excitatory role in promoting and maintaining wakefulness. This is in fact true whether the molecule is located in the intra-synaptic (95) or extra-synaptic space (96). Besides this physiological role, cellular glutamate metabolism has gained much interest recently due to its apparent crucial role in the survival of malignant cells, especially in their cellular growth and proliferation (97).

Glutamate is mostly secreted into the synaptic cleft by the cysteine-glutamate transporter (system x^-^_c_), which exchanges it with extracellular cysteine. Glutamate cannot passively diffuse back to the intracellular space, nor can it be metabolized by extracellular enzymes. It is therefore transported into the intracellular space primarily by molecules known as the excitatory amino acid transporters (EAATs) (98). Glutamate release and uptake to and from the synaptic cleft are both tightly regulated through the aforementioned molecules. If this tight control is disrupted, it leads to glutamate accumulation and causes detrimental excitotoxicity (99,100). This type of damage can also occur in the presence of necrosis, which causes the intracellular glutamate to leak into the extracellular space, damaging cells, and causing a cascade of neurotoxocity (100) and further cellular decay (101). This over-abundance of extracellular glutamate is detrimental not only to the surrounding cells, but also to the cellular ability to cope with reactive oxygen radicals. This happens since the abundance of extracellular glutamate impairs the system x^-^_c_, which normally exchanges it with extracellular cysteine. The deficiency of this transporter leads to an intracellular lack of cysteine, which in turn causes a complex cascade in which cellular cysteine and subsequent glutathione production are impaired (100), therefore disabling the anti-oxidative properties of glutathione (see earlier).

The abundance of glutamate in malignant brain tumors is well-known (102-104). And even though in the past it has been proposed that this abundance is caused primarily by tumor cells necrosis (thus being merely a side-effect of necrosis), it has since been repeatedly shown that malignant cells express a significant upregulation of system x^-^_c_ as well as a significant downregulation of EAAT molecules. Both of these changes of genetic expression allow for a higher extracellular glutamate concentration (100). This would therefore imply an important role that extracellular glutamate has in the survival of malignant cells. Knowing the positive effect that disrupted sleep has on tumor cells, it seems likely that at least one of the functions of this active glutamate secretion is actually chemically disrupting sleep.

## Clinical implications

The currently standardized treatment of patients with malignant brain tumors consists of surgery, chemotherapy, and radiotherapy. Diagnosis and treatment of sleep disorders in these patients is seldom considered. As mentioned earlier, many researchers have already noticed this fact (4,8,10,28,30,105), thus we will not go into this issue in depth. Suffice to say that the cited articles include several methods on how to measure and diagnose sleep disorders, as well as how to treat them, with a special emphasis on the evaluation of treatable underlying causes of sleep disorders, as well as the importance of sleep hygiene alone or in addition to pharmacological management.

The fact that disrupted sleep is such a common symptom and so crippling to the quality of life of patients with malignant brain tumors is well known. Thus, the currently frequent disregard of the problem in the oncological treatment increasingly seems insensible and misguided. The added benefit of actively recognizing and addressing this problem is that it has at least some potential in actively suppressing the vicious disease. We would therefore encourage all centers treating patients with malignant brain tumors to actively include somnologists into the multidisciplinary teams, and to actively address the disrupted sleep that these patients likely suffer from.

## Conclusions

In this article, we tried to raise two main issues. The first one is that considering the current knowledge of the relationship between sleep disruption and malignant diseases, it is quite possible that disrupted sleep is not just a common symptom but actually a possible factor in the disease progression. We thus argue that addressing the issue in patients with malignant brain tumors cannot only be a palliative measure that has the potential to improve the quality of life of these patients but can also prove to be a possible therapeutic approach in limiting the disease progression, especially when other treatment modalities have been exhausted. Worth noting however, is that all of this research is still in its theoretical stage, and treatment of sleeping schedules should not be considered as a therapeutic approach against a malignant brain tumor until further research is conducted. The second issue we tried to raise is that patients with malignant brain tumors are especially susceptible to these impaired sleeping schedules. Not just because of the oncological treatment they receive, but also due to the innate properties of malignant cerebral lesions and their specific interactions with the healthy brain. Indeed, we feel that this area of interest is currently underexplored and merits further research into the subject.
